# In-Situ Rheological Studies of Cationic Lignin Polymerization in an Acidic Aqueous System

**DOI:** 10.3390/polym12122982

**Published:** 2020-12-14

**Authors:** Samira Gharehkhani, Weijue Gao, Pedram Fatehi

**Affiliations:** Green Processes Research Centre and Biorefining Research Institute, Lakehead University, Thunder Bay, ON P7B5E1, Canada; sgharehk@lakeheadu.ca (S.G.); wgao@lakeheadu.ca (W.G.)

**Keywords:** lignin polymerization, rheology, particle size, biorefining

## Abstract

The chemistry of lignin polymerization was studied in the past. Insights into the rheological behavior of the lignin polymerization system would provide crucial information required for tailoring lignin polymers with desired properties. The in-situ rheological attributes of lignin polymerization with a cationic monomer, [2-(methacryloyloxy)ethyl] trimethylammonium chloride (METAC), were studied in detail in this work. The influences of process conditions, e.g., temperature, component concentrations, and shear rates, on the viscosity variations of the reaction systems during the polymerization were studied in detail. Temperature, METAC/lignin molar ratio, and shear rate increases led to the enhanced viscosity of the reaction medium and lignin polymer with a higher degree of polymerization. The extended reaction time enhanced the viscosity attributing to the larger molecular weight of the lignin polymer. Additionally, the size of particles in the reaction system dropped as reaction time was extended. The lignin polymer with a larger molecular weight and R_g_ behaved mainly as a viscose (tan δ > 1 or G″ > G′) material, while the lignin polymer generated with smaller molecular weight and shorter R_g_ demonstrated strong elastic characteristics with a tan (δ) lower than unity over the frequency range of 0.1−10 rad/s.

## 1. Introduction

Lignin, the largest reservoir of aromatic compounds on Earth, has attracted tremendous interest [1,2,3]. The annual production of lignin, mainly from the pulping industry, has been reported to be more than 50 million tons [4]. Particular interests have been placed in tailoring lignin’s characteristics through chemical reactions and polymerization with other components [5]. Monomers with altered structures were polymerized with lignin to produce water-soluble lignin-based polymers [2,6,7,8,9,10]. For example, the polymerization of N-isopropyl acrylamide (NIPAM) with hardwood kraft lignin was reported [11]. Our previous studies introduced a new process for inducing a cationic lignin-graft-[2-(methacryloyloxy)ethyl] trimethylammonium chloride (METAC) polymer under the optimized polymerization conditions of pH 4.0, METAC/lignin molar ratio 1.8, 3 h, 80 °C, lignin concentration of 0.3 mol/L, which resulted in the charge density of 2.93 meq/g and the grafting ratio of 178.5% [3,12]. Despite extensive studies on the chemistry and optimization of the polymerization reaction, limited attention was dedicated to the rheological and viscosity variations in the polymerization reactions. Despite extensive studies on the chemistry and optimization of the polymerization reaction, limited attention was dedicated to the rheological and viscosity variations in the polymerization reactions.

The rheological characteristics of a polymer in solution depend on its structure. During the polymerization reaction, the alteration in the structure of the polymer, e.g., molecular weight, leads to the variation in its rheological behavior in solution [13]. To investigate how the polymerization can be controlled for tailoring polymers with desired properties, monitoring the rheological behavior of the polymerization process is crucial. In addition, monitoring the rheological characteristics of the polymerization process would provide invaluable information for the design of equipment, e.g., pump and impeller, required for the large-scale production of polymers [14,15]. 

Several rheology studies were reported for the polymerization and cross-linking of different chemicals [16,17,18,19]. Gimenez studied the rheological characteristics of the polymerization of α-caprolactone [20]. The author presented a model to predict the evolution of the rheological properties of reactive systems in bulk polymerization [20]. Another model was also presented by Bae and coworkers for the viscosity of epoxy/phenol resin mixtures during the curing reaction [21]. An analysis of rheokinetic data obtained during the formation of functional polyurethane showed that the viscosity of the system would increase with curing time exponentially [22]. In another research carried out by Vulpe [23], an in-situ rheology study was performed on chemically crosslinked hydrogels made of collagen, hyaluronic acid, and sericin to understand the molecular dynamics of crosslinking mixtures and the kinetics of the crosslinking process. Additionally, the rheokinetic studies of the graft polymerization of acrylamide in a concentrated starch solution were performed by Bao and coworkers to understand how the initiator concentration and reaction temperature would impact the reaction progress [24]. These examples demonstrated the advantage of using in-situ rheology studies in understanding the polymerization systems, which would also be important for evaluating lignin polymerization systems. Generally, most of the in-situ rheology studies were performed using parallel plate geometry and through oscillation experiments at a low strain without considering the factor of mechanical mixing [21]. As mechanical stirring is an established method in the polymerization processes, the present study focused on understanding how the shear flow would impact the viscosity evolution of the cationic lignin polymerization process.

In the present work, we elucidated the rheological behavior and structure of the lignin polymer during its polymerization reaction. To the best of the authors’ knowledge, despite the numerous studies on lignin melt rheology, there is no information on the rheology of lignin polymerization systems. Considering our previous successful efforts on cationic lignin polymerization, [2-(methacryloyloxy)ethyl] trimethylammonium chloride (METAC) was selected to render lignin cationic [6,12,25].

Cationic flocculants have widely been applied in water treatment, such as sludge dewatering and the removal of other negatively charged colloidal particles [12,26,27], and our previously produced cationic lignin-METAC polymer showed promising results [6,12,25]. For example, the cationic lignin-METAC polymer was an effective flocculant for removing anionic azo-dyes of reactive black 5 (>98%) and reactive orange 16 (94%) [28]. The flocculation performance of the lignin-METAC polymer was also evaluated in a 0.25 wt % kaolin suspension using a particle dispersion analyzer, and the results demonstrated better flocculation efficiencies obtained for the lignin-METAC polymer than PMETAC and unmodified lignin [12]. However, the commercially available flocculants are primarily synthetic polymers, which are non-biodegradable and sometimes toxic [27,29]. It is industrially attractive to develop a green process for the production of a sustainable product (e.g., lignin-based cationic flocculants). The formation of the high molecular weight of lignin-METAC during the polymerization process would significantly affect the rheological behavior of the reaction mixture and the flocculation characteristics of lignin-METAC products. Therefore, the influence of process conditions, such as temperature, time, monomer concentrations, and shear rates, on the viscosity evolutions of the reaction medium was explored in this study. 

The main products (LM) polymerized under different conditions were characterized for their molecular weight, charge density, and grafting ratio, and the relationship between viscosity variations, and the chemical characteristics of the lignin sample were investigated. Studies on the microstructure of reactant products were also included in the scope of the present work. This study is of interest to polymerization reaction monitoring, scale-up, and process control of such a reaction owing to the knowledge it creates about the polymerization mechanism and behavior of lignin at different stages of the polymerization reaction. The rheological behavior of polymerization reaction and the rheological behavior of the lignin polymer, after its production, were the topics of this work.

## 2. Materials and Methods 

### 2.1. Materials 

Softwood kraft lignin (KL) with a 20% moisture content was received from FPInnovations. [2-(Methacryloyloxy)ethyl] trimethylammonium chloride solution (METAC, 80 wt % in H_2_O), potassium persulfate (K_2_S_2_O_8_), 4-hydroxybenzoic acid, sulfuric acid (H_2_SO_4_, 98%), hydrochloric acid, sodium hydroxide (NaOH, 97%) and deuterium oxide (D_2_O) were purchased from Sigma-Aldrich (Markham, ON, Canada). All chemical reagents were used as received. Ethanol (95 vol %) and anionic polyvinyl sulfate (PVSK) were purchased from Fisher Scientific and Wako Pure Chem. Ltd., Japan.

### 2.2. Synthesis of Lignin-graft-poly METAC (LM)

To produce LM, we adapted a procedure that was developed in our group earlier [12]. Briefly, KL powder (2 g) was mixed in water (30 mL) and poured into a three-neck glass flask. After 20 min of stirring and purging the sample with nitrogen, METAC was added to the sample and the pH of the sample was adjusted to 3.5 using H_2_SO_4_, and the system was stirred for 20 min in a nitrogen environment. In the next step, 3.2 mL of an initiator (K_2_S_2_O_8_) with a concentration of 1 wt % was added to the mixture, and the final volume of the sample was adjusted to 40 mL. The sample was then transferred to a hybrid rheometer (TA Instruments, Discovery HR-2, New castle, DE, USA) with vane geometry (bob diameter of 28 mm and length of 42 mm) that was preheated to the desired temperature (60−80 °C). The polymerization process was taken place at different temperatures and for altered durations. To investigate the effects of the METAC/KL molar ratio, temperature, and shear rate on viscosity evolutions of the reaction media, the polymerization processes were conducted for 10000 s. For studying the time effect, various samples were polymerized at altered reaction times ranging from 500 to 10,000 s. To stop the reactions, the temperature of the rheometer dropped to room temperature. The purification process was performed by transferring the sample from the rheometer to a beaker containing 200 mL of ethanol (80 wt %). The LM product was obtained by centrifuging the suspension at 3500 rpm for 10 min followed by freeze-drying the samples.

### 2.3. Rheology Settings

#### 2.3.1. Flow Test 

In the first set of experiments, the polymerization was monitored inside the rheometer through the flow peak tests conducted at different temperatures and shear rates. In these tests, a constant shear rate (385 s^−1^ or 130 s^−1^) was applied to the sample inside the geometry and viscosity changes were recorded. 

#### 2.3.2. Reaction Product 

In one set of experiments, the microstructures of two sets of reaction products, which were obtained from (i) long reaction time (LM-1) and (ii) short reaction time (LM-2), were monitored using the oscillatory tests [24]. The experiments were performed using a parallel-plate geometry (stainless steel with a diameter of 40 mm) equipped with the solvent trap to minimize water evaporation. When the reaction was stopped, approximately 1 mL of reaction products were loaded onto the lower plate of the geometry. The experiments were conducted at room temperature (22 °C) and the gap between plates was set to 500 μm. An amplitude sweep test ranging 0.1−100% was conducted with an angular frequency of 10 rad/s to identify the linear viscoelastic region (LVR). Frequency sweep measurements were then carried out in the range of 0.1 and 100 rad/s with a strain of 10% chosen from the linear viscoelastic region.

### 2.4. Structural Characterization of LM

#### 2.4.1. Fourier Transform Infrared (FT-IR) 

The FT-IR spectra of LM were recorded using a Fourier transform infrared spectrophotometer (Bruker Tensor 37, Germany, ATR accessory) with a transmittance mode in the range of 600−4000 cm^−1^ with a 4 cm^−1^ resolution and 32 scans per sample. 

#### 2.4.2. Water Solubility Analysis

For solubility analysis of LM, 0.1 g of dried LM product was suspended in 10 g of deionized water and the pH of the sample was adjusted to 7 using NaOH followed by stirring overnight at room temperature. The suspension was then centrifuged at 3500 rpm for 5 min, and the amount of LM in the supernatant was determined after drying at 60 °C.

#### 2.4.3. Hydroxyl Group Content Analysis for LM-1 and LM-2

A potentiometric titration technique was employed to determine the phenolic hydroxy group contents of samples using an automatic potentiometer titrator (785 DMP Titrino, Metrohm, Switzerland). In a typical procedure, a solution was obtained by adding 0.06 g of synthesized sample into 100 mL of deionized water containing 1 mL of potassium hydroxide (0.8 mol/L) while stirring at 150 rpm for 10 min. Then, 4 mL of 4-hydroxybenzoic acid (0.5%), as an internal standard, was added to the solution. The titration was subsequently carried out against 0.1 mol/L of hydrochloric acid. The hydroxyl group content of the samples was calculated according to Equation (1):

(1) $$\mathrm{Phenolic}\;\mathrm{hydroxyl}\;\mathrm{group}\;\left(\frac{\mathrm{mmol}}{g}\right)=\;\frac{C_{\mathrm{HCl}}\left[\left(V_{2}^{\prime }-V_{1}^{\prime }\right)-\left(V_{2}-V_{1}\right)\right]}{m},$$

where C is the titrant concentration (HCl, 0.1 mmol/L), m is the mass (g) of the sample used for analysis, and V′_1_ and V′_2_ represent the HCl volume (mL) used at first and second endpoints when LM solutions were titrated. Additionally, V_1_ and V_2_ are the HCl volume (mL) used at two endpoints during the titration of the blank solution. Moreover, m represents the weight (g) of lignin samples.

#### 2.4.4. Unreacted Lignin and Monomer for LM-1 and LM-2

The supernatants separated from the ethanol washing/centrifuging process were considered for determining the amount of unreacted lignin and monomer. T amounts of unreacted lignin in LM-1 and LM-2 samples were measured by the UV–Vis spectrophotometer (Thermo Scientific, Madison, WI, USA) at 280 nm. To measure the unreacted METAC, a procedure described by Wang and coworkers was followed [12]. Briefly, the total mass concentration of the supernatants (m_1_, g/L) was obtained by drying the supernatant in an oven at 105 °C for 48 h. One part of the same supernatant was then dialyzed for 48 h to eliminate unreacted METAC. The supernatant was dried in an oven and the concentration of the supernatant was determined (m_2_). The percentage of unreacted METAC was then evaluated using Equation (2):

(2) $$\mathrm{Percentage}\;\mathrm{of}\;\mathrm{unreacted}\;\mathrm{METAC}=\frac{m_{1}-m_{2}}{m}\times 100\%$$

where m (g) is the original concentration of METAC, m_1_ (g/L) is the total mass concentration of the supernatant after the reaction, and m_2_ is the mass concentration of the supernatant after removing unreacted METAC.

#### 2.4.5. Elemental Analysis

An elemental analyzer (Vario EL, Straße 1, Germany) was used to determine the nitrogen contents of samples. The organic elements of the samples were obtained by combusting 0.05 g of oven-dried samples at 1200 °C. An earlier study showed that the nitrogen content of the sample could be used to calculate the extent of grafting ratio using Equation (3):

(3) $$\mathrm{Grafting}\;\mathrm{ratio}\;\left(\mathrm{wt}.\;\%\right)=\;\frac{N\%/14\times M_{W}}{100-N\%/14\times M_{W}}\times 100$$

where N (wt %) is the nitrogen content of samples and M_W_ is the molecular weight of METAC (172.3 g/mol).

#### 2.4.6. Charge Density

First, 0.1 g of dried samples were dispersed in 10 mL of distilled water and stirred overnight at 100 rpm for 1 h at 30 °C in a water bath shaker (Innova 3100, Brunswick Scientific, Edison, NJ, USA). The samples were then centrifuged at 1000 rpm for 5 min and the supernatants were decanted for analysis using a particle charge detector, Mütek PCD 04 titrator (Arzbergerstrae, Herrsching, Germany). The titration was performed against a PVSK standard solution (0.005 mol/L).

#### 2.4.7. Molecular Weight Analysis

A solution containing 3 mg of freeze-dried LM and 10 mL of 5% acetic acid was prepared for the test. After overnight stirring, the solution was filtered using a 0.2 μm syringe filter. To determine the molecular weight, the solution was injected into a gel permeation chromatography system, Malvern GPCmax VE2001 Module, Cambridge, UK, and Viscotek TDA305 with a multidetector. PolyAnalytic PAA206 and PAA203 columns were used for this set of experiments. Flow rate and column temperature were adjusted to 0.70 mL/min and 35 °C, respectively.

#### 2.4.8. Particle Size Analysis

A laser diffraction particle size analyzer (Malvern Mastersizer 3000, Worcestershire, UK) was used to detect the particle size of LM samples prepared at various reaction times. To perform the analysis, a mixture containing purified freeze-dried LM (concentration of 1 wt %) was prepared. After 3 h of stirring at room temperature, 1 mL of sample was added into a water chamber. To break down the agglomerations of LM, an ultrasound with a power of 60% for 30 s followed by stirring at 2000 rpm was applied in the measurements.

#### 2.4.9. Radius of Gyration (R_g_)

A static light scattering (SLS) instrument attached to a goniometer, Brookhaven BI-200SM, Holtsville, NY, USA was used to measure the R_g_ of purified LM-1 and LM-2 samples. Five different concentrations of lignin solutions (0.2, 0.4, 0.6, 0.8, and 1.2 wt %) were prepared by dissolving the samples in 0.75 M NaOH at room temperature. The solutions were then filtered using a nylon syringe filter (30 mm diameter and 0.45 μm pore size). The wavelength of laser polarized light was 637 nm, and the intensities of different samples were tested at different angles (15−155°). The R_g_ of the samples was then determined using BIC Zimm Plot software.

## 3. Results and Discussions

### 3.1. Polymerization Mechanism

The chemistry of lignin polymerization with METAC was discussed in our previous work [12]. The polymerization of lignin and METAC is performed through free radical polymerization (Figure S1 in supplementary material). Softwood KL is mainly guaiacyl lignin, which is composed of coniferyl alcohol units [30]. In this polymerization reaction, the monomer reacts with both the aliphatic and phenolic hydroxyl groups of lignin generated by the sulfate radicals [12,25,31]. The grafting of the METAC segment carrying ammonium groups onto the lignin structure affords a water-soluble cationic LM [12]. Although the structure of lignin depends on its plant sources and the methods for lignin extraction/production from biomass, all lignin subunits (e.g., p-hydroxyphenyl (H), guaiacyl (G), and syringyl (S)) contain aliphatic and phenolic hydroxyl groups, which are involved in this polymerization reaction. Therefore, in addition to kraft lignin, this polymerization reaction could potentially be applied to other types of lignin, e.g., hydrolysis lignin, alkali lignin, organosolv lignin, and lignosulfonates. The FTIR spectrum of LM is shown in Figure S2 in the supplementary material, which shows the characteristic peaks of LM and confirmed the success of the reaction [12].

### 3.2. Effect of Process Conditions on Viscosity Evolution during Polymerization

#### 3.2.1. Effect of Temperature

**Figure 1:**
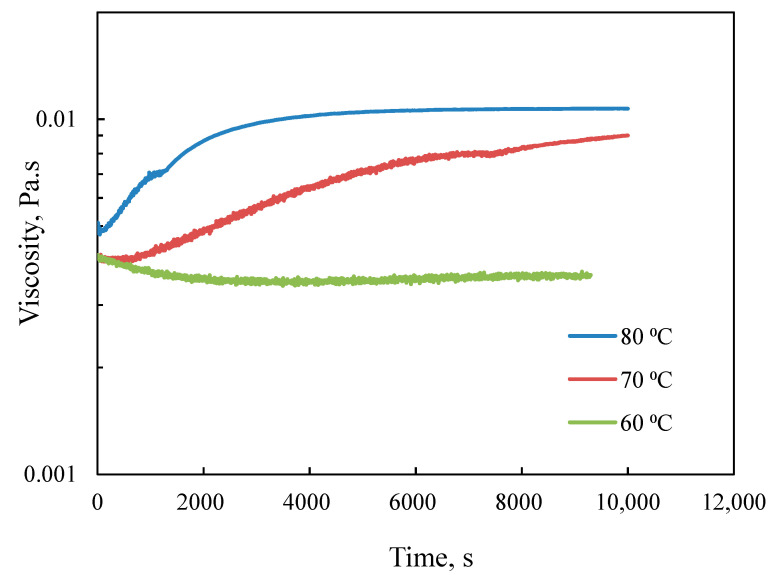
Variation in the viscosity of the reaction products as a function of time in the polymerization of METAC/lignin with a molar ratio of 2.3 at different temperatures (60, 70, and 80 °C).

Process conditions, such as reaction temperature, ratio, and concentration of reagents and shear rates, could influence the physicochemical characteristics of the resultant polymerization system. Figure 1 shows the effect of temperature on the viscosity of the reaction medium during the polymerization process. Data depicted that the temperature has a substantial effect on the viscosity. At 60 °C, the viscosity of the medium was almost constant during the polymerization process. At a higher temperature, the viscosity increased sharply and then reached a constant value. The temperature of the polymerization process can influence the reactivity of monomers and increase the polymer’s radical concentration and mobility where an increase in the viscosity can be interpreted as the progress in polymerization. This hypothesis was later confirmed through the characterization of samples. Moreover, an increase in the temperature resulted in a sharp increase in the viscosity, specifically at 80 °C and 70 °C. These results are consistent with a previous study on the free radical polymerization where a faster increase in the dynamic modulus was observed during the graft polymerization of acrylamide in a concentrated starch medium [24].

#### 3.2.2. Effect of Molar Ratio

**Figure 2:**
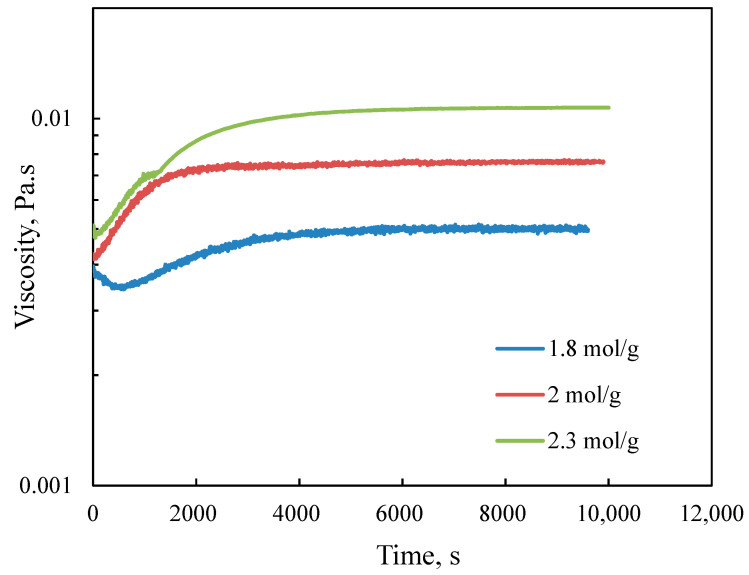
Variation in the viscosity of the reaction medium as a function of time in the polymerization of METAC/lignin with different molar ratios at 80 °C.

The concentration of reactants can strongly affect the characteristics of the polymerization product. Figure 2 illustrates the effect of the METAC/lignin molar ratio on the viscosity evolution of the reaction medium. The higher the molar ratio, the more viscous the reaction medium was. A raise in the METAC load provided more interactions between lignin and METAC, resulting in a more viscous mixture corresponding to a higher yield of the LM product. 

#### 3.2.3. Effect of Shear Rate

**Figure 3:**
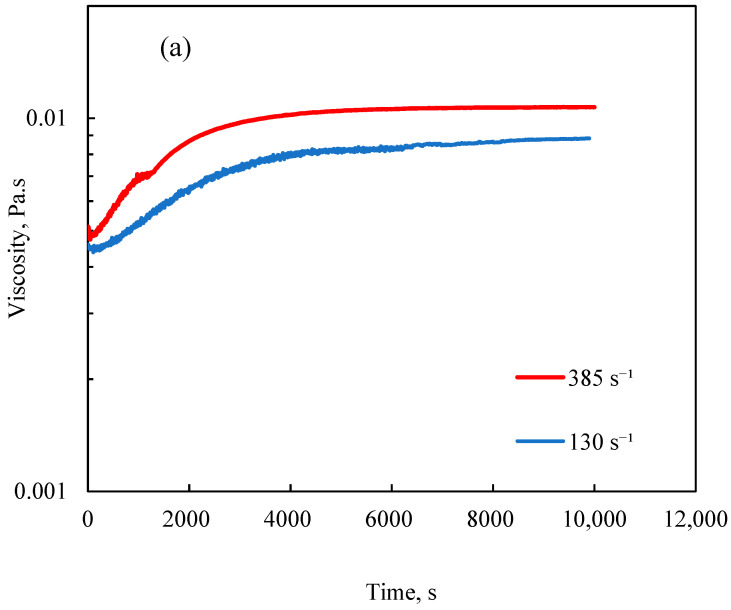

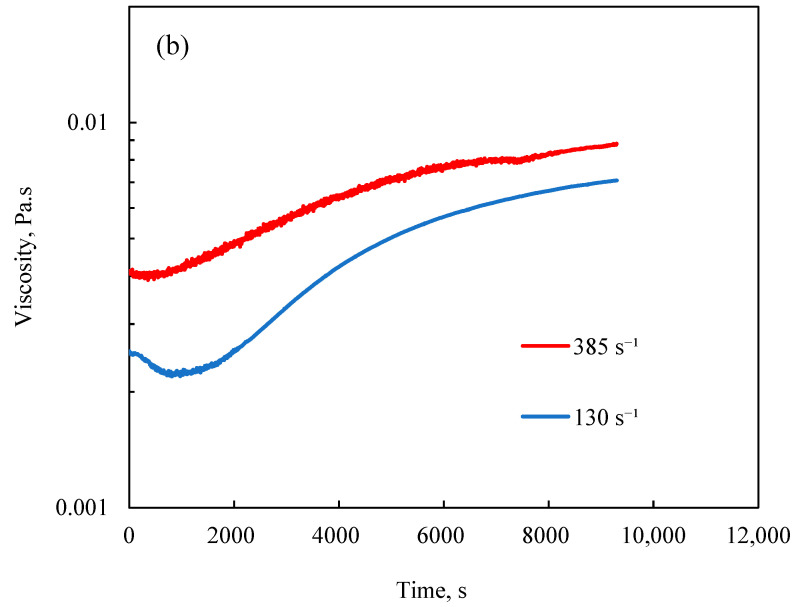
Variation in the viscosity of the reaction medium as a function of time in the polymerization of METAC/lignin at different shear rates for samples polymerized at a temperature of (**a**) 80 °C and (**b**) 70 °C at the METAC/lignin molar ratio of 2.3.

The effect of shear rate, as a representative of agitation intensity, on the polymerization of LM was also investigated at two different temperatures (Figure 3a,b). The two different shear rates of 130 and 385 s^−1^ were corresponded to the rotations of 100 and 300 rpm, respectively. Regardless of the temperature, a decrease in the shear rate resulted in a decrease in viscosity, and the change was more pronounced at 70 °C. Moreover, a progressive increment in the viscosity was seen at both shear rates at 70 °C, implying that the reaction probably needed a longer time to develop.

### 3.3. Characteristics of Cationic Polymers

Table 1 presents the characteristics of samples polymerized at various conditions in terms of the molecular weight, charge density, and grafting ratio. Lignin utilized in the present study contained a very low nitrogen content, while METAC carrying ammonium groups had a considerable amount of nitrogen. Therefore, the nitrogen content of LM was used in evaluating the grafting ratio. It is seen that LM-1, which was polymerized at 80 °C with the shear rate of 385 s^−1^ and METAC/KL ratio of 2.3 posed the highest molecular weight, charge density, and grafting ratio. The lowest amounts of grafting ratio (41.5%) and charge density (1.4 meq/g) were obtained for the sample polymerized at 60 °C. The results also presented that the decrease in the METAC/KL molar ratio and shear rate resulted in polymerized LM with a lower grafting ratio. It is notable that at a specific temperature, the most important factor in altering the LM samples was the METAC/KL molar ratio, where an increase in the molar ratio resulted in an improvement in LM properties. An increase in the molar ratio from 1.8 to 2.3 mol/g led to an enhancement of 93.3%, 52.0%, and 72.5% in molecular weight, charge density, and grafting ratio, respectively (Table 1). According to these results, it can be concluded that the viscosity development in the polymerization was significantly affected by the METAC/KL molar ratio, temperature, and shear rate due to their influences on the molecular weight, grafting ratio, and charge density.

### 3.4. Insights into LM Characteristics during Polymerization

**Figure 4:**
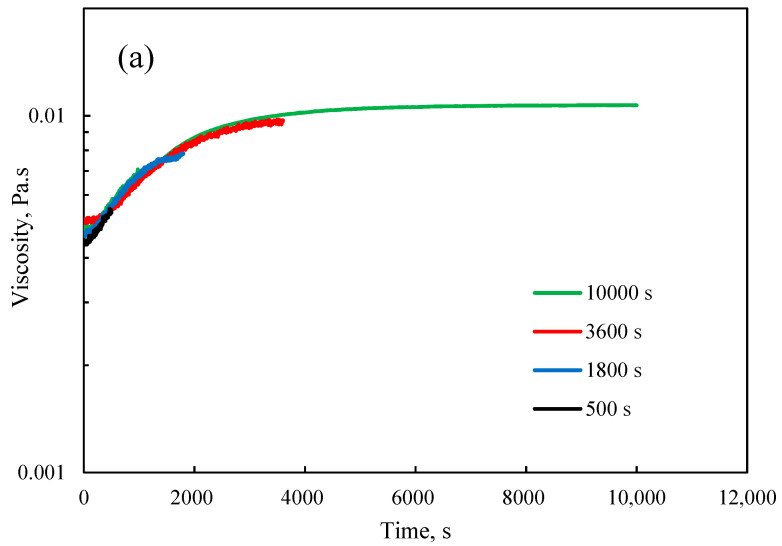

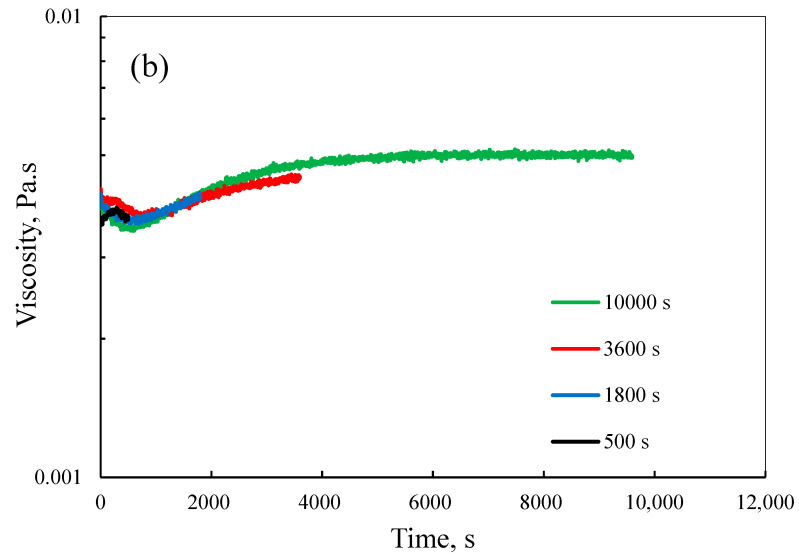
Viscosity of the reaction medium as a function of time for samples prepared at temperature 80 °C, and METAC/lignin molar ratios of (**a**) 2.3 and (**b**) 1.8 mol/mol.

To gain insights into the relationship between the viscosity and polymerization progress, a set of experiments was conducted by quenching the reactions at various reaction times. Figure 4a,b illustrate the viscosity evolutions of the samples with different METAC/lignin molar ratios conducted at various reaction times. All graphs followed a similar trend implying a good reproducibility of data. The characteristics of samples tabulated in Table 2 depicted the progress in the polymerization as a function of prolonged reaction time. For example, the grafting ratios for samples polymerized with the METAC/KL molar ratios of 1.8 and 2.3 after 500 s were 27.4% and 59.8%, respectively, while the reaction after 10,000 s yielded the products with 79.5% and 137.2% grafting ratios.

**Figure 5:**
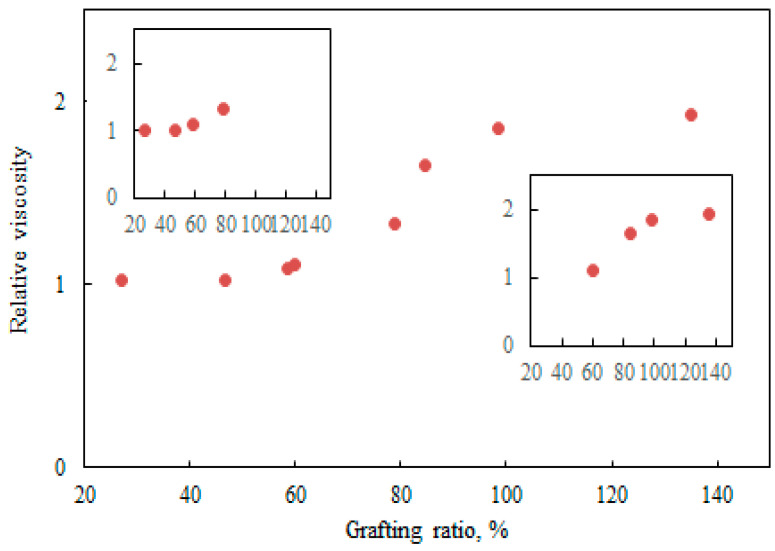
Relationship between relative viscosity and the grafting ratio.

To find out a relationship between the polymerization progress and viscosity of the reaction medium, data presented in Figure 4 and Table 2 were used in determining the relative viscosity (η_final_/η_0_) of the samples as a function of the grafting ratio in Figure 5. The relative viscosity was used as a dimensionless parameter to reduce the variables. In this figure, a three-stage polymerization can be identified. In the first stage, the relative viscosity was almost constant for a low range of grafting ratios, which were resulted from a low degree of polymerization. Along with the progress in the polymerization, the relative viscosity was increased in the second stage. The enhanced relative viscosity is attributed to the increase in the molecular weight of polymer chains [21]. In the third stage, the relative viscosity changed insignificantly. At this stage, the high grafting ratio could lead to the critical concentration above which macromolecules in the solution began to touch each other and the relative viscosity became independent of molecular weight [32,33]. As shown in Figure 5, the first stage in the relative viscosity variations corresponded to the data recorded for the low amount of METAC in the reaction, while the data obtained from the high METAC/KL molar ratio covered the other two stages. Li et al. also found that during the grafting of methyl acrylate (MA) monomers onto native sesbania gum (SG), a higher grafting ratio led to the higher apparent viscosity of SG-g-PMA paste as the increases in the number and the length of grafted branches increased the molecular weight of SG-g-PMA [34]. 

### 3.5. Microstructure of Reaction Products

The information about the microstructures of reaction products was also obtained through another series of experiments performed on LM-1 and LM-2 reaction products. The characteristics of samples were examined in the linear viscoelastic region (LVR; Figure S3 in the supplementary material), where the LVR was similar for both samples. 

**Figure 6:**
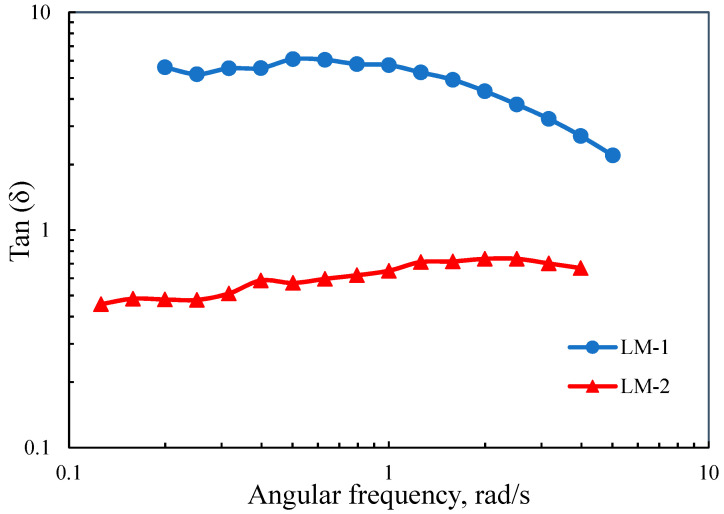
Variation in tan (δ) as a function of angular frequency.

The variation of tan (δ) over a frequency range is presented in Figure 6. Tan (δ) is defined as G″/G′ presenting the ratio of energy lost through viscous dissipation in a cycle of deformation to the magnitude of the energy stored in the elastic structure [35−37]. These dynamic shear moduli (G′ and G″) are defined as G′ = σ_0_ cos(δ)/γ_0_ and G″ = σ_0_ sin(δ)/γ_0_. 

**Figure 7:**
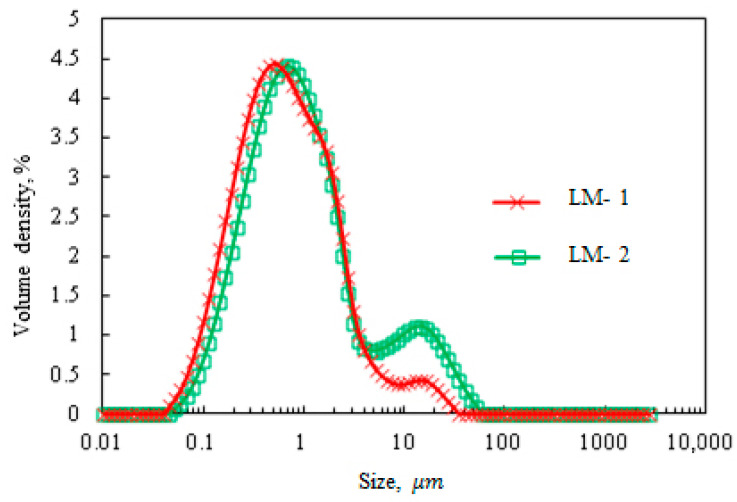
Size distribution of LM-1 and LM-2.

For pure solid and liquid states, δ is equal to 0° and 90°, respectively [38]. In classical viscous fluids, the dynamic moduli are frequency-dependent where G″ >> G′ [39]. In the present study, the LM-1 sample behaved mainly as a viscose (tan δ > 1 or G″ > G′), indicating that the entanglements between medium components are not strong and have sufficient time to disentangle [23]. Unlike LM-1, LM-2 had a tan (δ) lower than unity over the frequency range, demonstrating strong elastic characteristics. These observations in the present study can be explained by the change in lignin structure during polymerization and the interactions between reaction components. The size of lignin polymer aggregations is one of the factors. Figure 7 illustrated that the progress in polymerization triggers aggregations. For both samples, a bimodal distribution was observed with a peak near 0.6 μm and another one at approximately 18 μm. For a short reaction, the presence of large particle clusters can interconnect and reflect more elastic properties. As the reaction progressed, the substitution of hydroxyl groups with amine groups made lignin more water-soluble (see Table 3), in which LM-1 can form smaller aggregates than did LM-2. 

Extended reaction time led to higher molecular weight (Table 2) and thus longer polymer chains. The longer the polymer chain, the higher the polymer flexibility would be. Improved flexibility within the cross-linked epoxy-amine network through the utilization of an amine reactant with a longer chain length was also reported by Nakka et al. [40]. The sample with these flexible chains (LM-1) formed less entanglement than the LM-2 having short polymeric chains [41,42]. The viscoelastic behavior of LM-1 and LM-2 can also be explained based on their R_g_ values. The R_g_ of a polymer is a geometrical quantity defined as an average square distance of the chain segments from the center of the mass of the chain of a polymer segment [43]. It has been stated that the R_g_ is not only proportionally related to the chain length, the conversion of functional groups, and consequently molecular weight but also represents the molecular shape of the polymer [44,45]. The polymer with smaller R_g_ reflects more a compact structure. Lobanov et al. [46] studied the dependency of protein pack structure on the R_g_ and observed that protein with smaller R_g_ had a more compact structure. The larger R_g_ of LM-1 (34.3 nm) than LM-2 (25.4 nm) indicated that the progress in the polymerization can change the conformation of lignin molecules toward a looser molecular shape, in which this new conformation posed more viscose characteristics (Figure 6).

**Figure 8:**
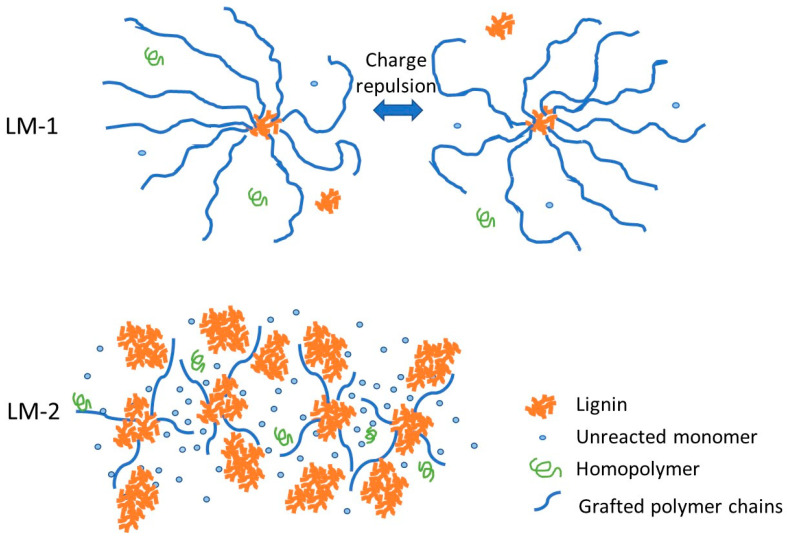
Schematic representing the configuration of reactant products for long (LM-1) and short (LM-2) time reactions.

Apart from the influence of lignin structural changes on viscoelastic properties, the interaction between reaction components should be considered. These interactions may include lignin-monomer/homopolymer interactions, which were shown in Figure 8. In the reptation theory, the mobility of flexible polymer chains in a crosslinked network will be restricted by the unattached components as the obstacles preventing the random motions of the chains [47,48]. For LM-2, as the reaction was not complete, the amounts of unreacted components, e.g., unreacted lignin and monomers, were high in the medium compared to the LM-1 (see Table 3). These unreacted elements would act as barriers to the movement of polymer chains. Therefore, due to the presence of more obstacles, the movement of polymer chains in LM-2 sample was more restricted, consequently, the reaction medium presented solid-like behavior. Moreover, in the case of LM-1 sample with a high degree of polymerization and great charge density (Table 2 and Table 3), the electrostatic repulsion between like-charged polymer chains might contribute to its liquid-like characteristics.

## 4. Conclusions

The formation of the cationic lignin polymer was studied through in situ rheology experiments. Viscosity changes during the polymerization of KL and METAC were significantly affected by the METAC/KL molar ratio, temperature, and shear rate due to their influences on the molecular weight, grafting ratio, and charge density of the induced polymer. The temperature elevation from 60 to 80 °C led to an increase in the viscosity of the reaction medium. The higher METAC/lignin molar ratio yielded a more viscose reaction medium. A decrease in the shear rate lowered the viscosity. The time extension changed the behavior of the reaction medium to be more liquid-like behavior in a broader range of frequencies. The larger R_g_ of LM-1 than LM-2 indicated that longer polymerization changed the conformation of lignin molecules toward a looser molecular shape, which would facilitate the disentanglements between reaction components, leading to more viscose (tan δ > 1 or G″ > G′) behavior. Additionally, shorter reaction time afforded LM-2 samples with low solubility where interconnected larger aggregations were formed and exhibited a tan (δ) lower than unity over the frequency range, and thus the LM-2 induced more elastic properties. A smaller particle size was obtained for the LM-1 sample synthesized at 10,000 s due to the solubilization of lignin with a high degree of polymerization.

## Figures and Tables

**Table 1:** Characteristics of the samples polymerized under different conditions (reaction time = 10,000 s).

Sample	Temperature (°C)	METAC/Lignin Molar Ratio	Shear Rate (s^−1^)	Molecular Weight (Kg/mol)	Charge Density (meq/g)	Grafting Ratio (%)
LM-1	80	2.3	385	990.5	3.8	137.2
LM-3	70	2.3	385	625.8	3.1	98.9
LM-4	60	2.3	385	- *	1.4	41.5
LM-5	80	2	385	823.4	3.4	103.3
LM-6	80	1.8	385	512.4	2.2	79.5
LM-7	80	2.3	130	798.5	3.2	101.2
LM-8	70	2.3	130	524.1	2.9	84.3

* Molecular weight could not be determined because of poor solubility of the sample in the solvent used for gel permeasion chromatography

**Table 2:** Characteristics of LM generated at temperature 80 °C, the shear rate of 385 s^−1^, and different reaction times.

Molar Ratio of 1.8				Molar Ratio of 2.3		
Time s	Molecular Weight (kg/mol)	Grafting Ratio (%)	Charge Density (meq/g)	Molecular Weight (Kg/mol)	Grafting Ratio (%)	Charge Density (meq/g)
500	- *	27.4	1.0	- *	59.8	1.8
1800	- *	46.7	1.1	- *	84.5	2.0
3600	- *	58.5	1.6	520.7	98.5	2.5
10,000	512.4	79.5	2.2	990.5	137.2	3.8

* The molecular weight of the samples prepared were not provided due to the poor solubility of samples in acetic acid 5% used as eluent in the GPC analysis.

**Table 3:** Characteristics of LM-1 and LM-2 samples.

Sample	Unreacted Lignin (%)	Unreacted METAC (%)	Phenolic Hydroxyl Content (mmol/g)	Solubility (%)
LM-1	0.8	10.2	1.45	97%
LM-2	6.5	48.5	1.67	70%

## Data Availability

The raw data required to reproduce these findings cannot be shared at this time as the data also forms part of an ongoing study to commercialize the lignin polymerization process. Readers are encouraged to communicate with the corresponding author for more information.

## Supplementary Materials

The following are available online at https://www.mdpi.com/2073-4360/12/12/2982/s1, Figure S1: Scheme for polymerization of LM, Figure S2: FTIR spectra of LM product, Figure S3: Variation of complex modulus versus strain at frequency.
