# Supplementary material for: In-Situ Rheological Studies of Cationic Lignin Polymerization in an Acidic Aqueous System
Source: Polymers (Basel). 2020 Dec 14;12(12):2982. doi: 10.3390/polym12122982 (PMC7764959; doi:10.3390/polym12122982)
Supplement: Supplementary file 1 [file polymers-12-02982-s001.pdf]

Supporting Information

In-situ rheological studies of cationic lignin polymerization in an acidic aqueous system

Samira Gharehkhani, Weijue Gao, Pedram Fatehi*
Green processes research centre and Biorefining research institute, Lakehead University, Thunder Bay, ON, Canada, p7b5e1
*corresponding author: email: pfatehi@lakeheadu.ca, tel:807-343-8697

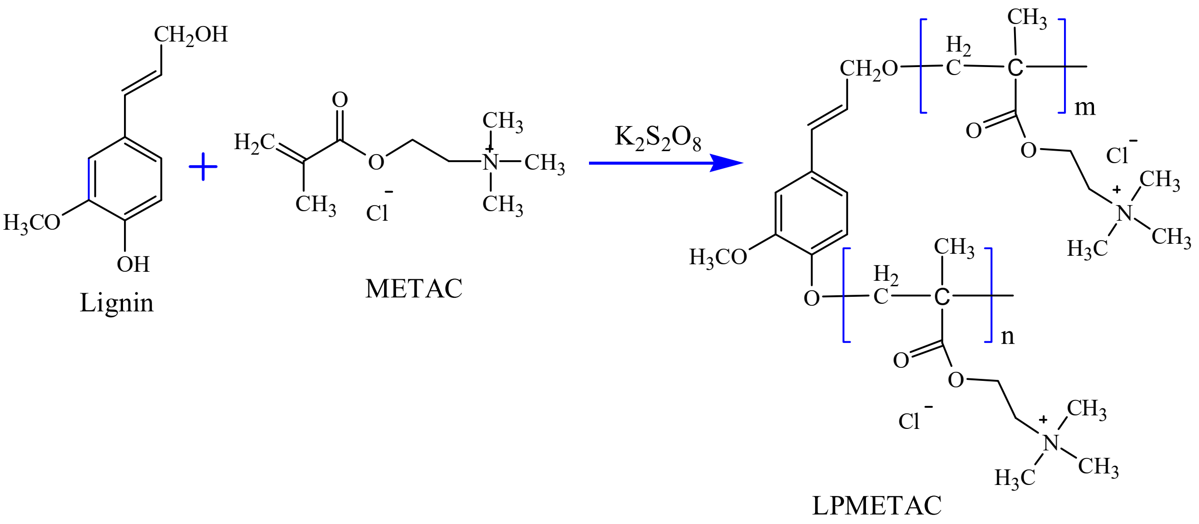

Fig. S1. Scheme for polymerization of LM

Fourier Transform Infrared (FT-IR) FT-IR spectra of LM and KL were recorded using a Fourier transform infrared spectrophotometer (Bruker Tensor 37, Germany, ATR accessory) with a transmittance mode in the range of 600−4000 cm^{-1} with a 4 cm^{-1} resolution and 32 scans per sample. Both LM and KL had a broad band at 3400 cm^{-1}, which was attributed to the stretch vibration of −OH groups. The spectra also depicted a band at 2900 cm^{-1}, corresponding to the C-H stretching in the methyl groups. The characteristic peaks of the aromatic skeletal vibration of KL were observed at 1591, 1510, and 1245 cm−1, respectively [12]. The new peaks appeared at 1722, 1477 and 960 cm-1 assigned to the C=O stretching vibration, C−N bending, and methyl groups of quaternary ammonium in PMETAC, respectively. These peaks did not appear in the spectrum of kraft lignin, implying the successful polymerization of lignin and METAC. The presence of these three-peak indicating the formation of LM polymer which is consistent with the previous study [12].

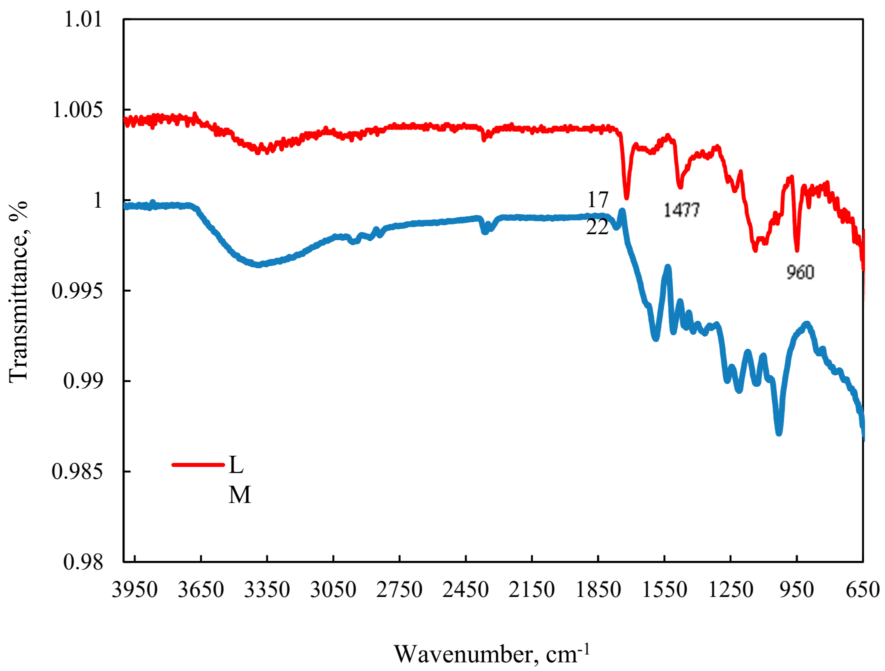

Fig. S2. FTIR spectra of LM product.

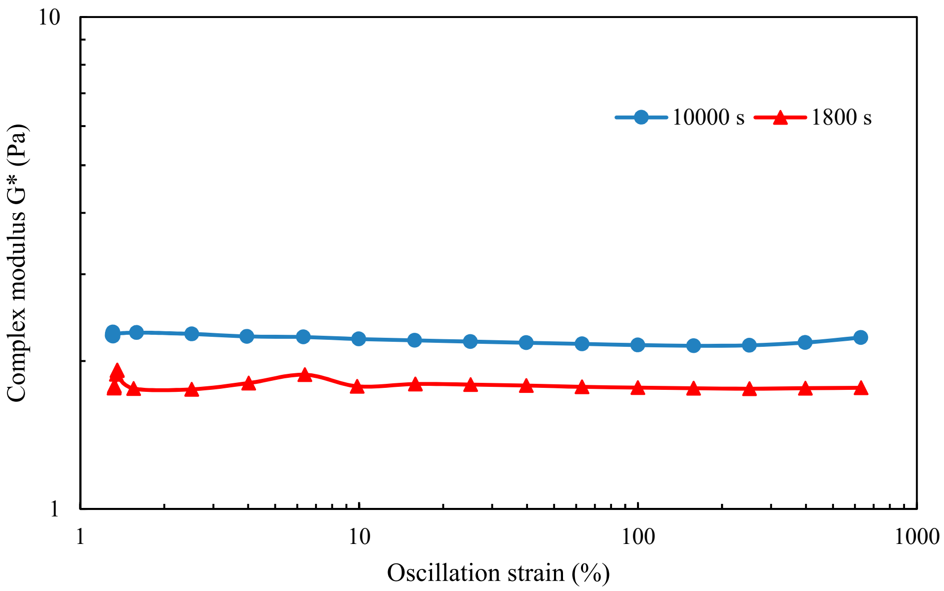

Fig. S3. Variation of complex modulus versus strain at frequency 10 rad/s for LM-1 (10000 s) and LM-2 (1800 s). The dynamic rheology experiments were conducted at RT.
